# QTL Mapping for Fiber and Yield Traits in Upland Cotton under Multiple Environments

**DOI:** 10.1371/journal.pone.0130742

**Published:** 2015-06-25

**Authors:** Hantao Wang, Cong Huang, Huanle Guo, Ximei Li, Wenxia Zhao, Baosheng Dai, Zhenhua Yan, Zhongxu Lin

**Affiliations:** National Key Laboratory of Crop Genetic Improvement, Huazhong Agricultural University. Wuhan 430070, Hubei, China; Institute of Cotton Research of Chinese Academy of Agricultural Sciences, CHINA

## Abstract

A population of 178 recombinant inbred lines (RILs) was developed using a single seed descendant from a cross between *G*. *hirsutum*. acc DH962 and *G*. *hirsutum*. cv Jimian5, was used to construct a genetic map and to map QTL for fiber and yield traits. A total of 644 polymorphic loci were used to construct a final genetic map, containing 616 loci and spanning 2016.44 cM, with an average of 3.27 cM between adjacent markers. Statistical analysis revealed that segregation distortion in the intraspecific population was more serious than that in the interspecific population. The RIL population and the two parents were phenotyped under 8 environments (two locations, six years), revealing a total of 134 QTL, including 64 for fiber qualities and 70 for yield components, independently detected in seven environments, explaining 4.40–15.28% of phenotypic variation (PV). Among the 134 QTL, 9 common QTL were detected in more than one environment, and 22 QTL and 19 new QTL were detected in combined analysis (E9). A total of 26 QTL hotspot regions were observed on 13 chromosomes and 2 larger linkage groups, and some QTL clusters related to fiber qualities or yield components were also observed. The results obtained in the present study suggested that to map accurate QTL in crops with larger plant types, such as cotton, phenotyping under multiple environments is necessary to effectively apply the obtained results in molecular marker-assisted selection breeding and QTL cloning.

## Introduction

Cotton (*Gossypium* L.) is an important economic crop, providing most of the natural textile fiber utilized worldwide. Upland cotton (*G*. *hirsutum*) is widely cultivated, and planted in more than 70 countries, contributing to over 95% of the total cotton yield worldwide [1]. Because of the softness and comfort of cotton fiber, cotton products are very popular. In recent decades, improvements in cotton fiber quality and yield have been stagnant and unable to meet the demands of the modern textile industry. However, yield is often negatively correlated with fiber quality in upland cotton [2,3]. Conventional cultivar breeding programs, primarily selecting novel allele combinations based on phenotypic selection [4], is difficult to break the linkage of negatively correlated traits. Fortunately, the development of genetic linkage maps facilitate the dissection of quantitative trait loci (QTL) that control fiber qualities and yield components, which make it possible to pyramid elite genes of fiber quality and yield traits.

Because of the low genetic polymorphism between the intraspecific hybridization of upland cotton, several high-density interspecific linkage maps between *G*. *hirsutum* and *G*. *barbadense* have been constructed to study QTL for fiber quality and yield traits [5–7]. However, due to the sterility and segregation distortion of interspecific progeny, intraspecific hybridization has become the primary method in breeding programs, contributing to the recent development of upland cotton intraspecific genetic maps [8–15].

As the most important cotton cultivar, much research attention has been paid to the improvement of the fiber quality and yield of upland cotton. The QTL mapping of fiber quality traits could provide a solid foundation for future studies concerning marker-assisted selection breeding and map-based cloning. Hundreds of QTL associated with fiber quality and yield components have been obtained from F_2_, F_2:3_, and RIL populations in upland cotton [8,9,12–25]. The F_2_ population is a common population used in genetic map construction and QTL mapping, but these studies cannot be replicated; thus, stable and available QTL could not be identified using the F_2_ population. The construction of immortalized mapping populations is an effective approach to obtain stable QTL, such as recombination inbred lines (RILs). However, the complex allotetraploid genome and agronomic traits in crops are inherited in a complex manner, suggesting that cotton traits are highly affected by environmental and climatic conditions, obtaining stable QTL in allotetraploid cotton is difficult. Tang et al. (2015) constructed a genetic map, containing 1,540 loci spanning 2,842.06 cM, and a total of 62 QTL were identified using combined analysis and single environment analysis; seventeen QTL were detected in more than one environment. Ning et al. (2014) identified 86 QTL for yield components and fiber qualities from an RIL population. In addition, a stable fiber strength QTL (qFS-D3-1), explaining 4.51–17.55% of the phenotypic variation (PV), and a stable fiber length QTL (qFL-D11-1), explaining 10.02–25.34% of the PV, were obtained.

In the present study, two upland cottons, DH962 and Jimian5, with different fiber qualities and yield component traits [21], were used as parents to establish a recombinant inbred line (RIL) population. The objectives of this study were to construct an intraspecific upland cotton map using SSRs, InDels and SNPs based on this RIL population, which was used to detect QTL associated with fiber quality and yield traits under multiple environmental conditions.

## Materials and Methods

### Mapping population and DNA isolation

The *G*. *hirsutum* acc. DH962 and *G*. *hirsutum* cv. Jimian5 were used as the mapping parents. DH962 was derived from the [(Jinmian6 × *G*. *thurberi*) F_4_ × Jinmian6] F_3_ population, showing good performance in fiber quality as a female parent and continuous self-pollination since 2001. Jimian5 is a cultivar with high yield as a male parent. DH962 and Jimian5 were crossed to obtain F_1_ plants on the farm at Huazhong Agricultural University (HAU), Wuhan, China, in the summer of 2002 [10]. The F_1_ plants were planted during the winter in Hainan Province and self-pollinated to produce the F_2_ generation. The F_2_ seeds were planted and self-pollinated to produce F_2:3_ seeds on the farm of HAU in 2003. An RIL population was developed using the single seed descendant method to generate F_2:7_ plants, which were subsequently planted at HAU for propagation in 2007. The F_7:8_ generations of 178 RIL families were used in the present study. Genomic DNA was isolated from the fresh leaves of 178 RIL plants and parents using a CTAB procedure [26].

### Fiber quality trait collection

The 178 RILs and parents were planted on a farm provided by Prof. Qizhong Xia at Huanggang Normal College, Huanggang (30.45° N, 114.93° E), Hubei, China in 2008 (E1), 2011 (E5), 2012 (E7) and 2013 (E8), and on a farm provided by Associate Prof. Dingguo Li at Yangtze University, Jingzhou (30.36° N, 112.15° E), Hubei, China in 2008 (E2), 2009 (E3), 2010 (E4) and 2011 (E6). These fields are only used for research purposes, and the field studies did not involve endangered or protected species. The fiber quality data in E7 and yield components in E1 were lost, and combined analysis (E9) was conducted after determining the mean values in seven environments. The lines were planted in single-row plots of 5 m in length with 0.8 m row spacing. All the lines were planted in the field using a randomized block. Twenty bolls from each line were simultaneously harvested for fiber quality and yield investigation. Six yield components and 5 fiber quality trait components were analyzed, including boll number per plant (BN), seed cotton weight per boll (SCW), lint weight per boll (LW), lint percentage (LP), lint index (LI), seed index (SI), fiber length (FL, mm), fiber strength (FS, cN/tex), fiber length uniformity ratio (FU), fiber elongation (FE), and micronaire (MIC).

### DNA marker analysis

A total of 634 SSRs, InDels, and SNPs, selected according to Wang et al. (2015) [15], were used to genotype each RIL plant. PCR amplification and silver staining were performed according to Lin et al. (2005) [27]. The SRAP markers were not genotyped in this RIL population because these polymorphisms are difficult to crosstalk with those identified in previous studies. The PCR products of SSRs were separated on 6% denaturing polyacrylamide gels [27] or 8% native polyacrylamide gels using single-strand conformation polymorphism (SSCP) technology [28]. The PCR products of InDels and SNPs were separated on 8% native polyacrylamide gels using SSCP technology [28].

### Data analysis, genetic map construction and QTL analysis

The difference between the two parents for each trait was detected using a t-test. The broad-sense heritabilities of measured traits were calculated according to the method of Knapp et al. (1985) [29]. The coefficients of genetic correlation between measured traits were computed according to the method of Kwon and Torrie (1964) [30]. The phenotype data were analyzed using SPSS version 21.0 (SPSS, Chicago, IL, USA).

A chi-square test was performed to determine whether the genotypic frequencies at each locus deviated from the expected 1:1 segregation ratio in the RIL population. The genetic linkage map of the RIL population was constructed using JoinMap 3.0 [31] with a logarithm of odds (LOD) threshold of 4.0 and a maximum recombination fraction of 0.4. Map distances in centi-Morgans (cM) were calculated using the Kosambi mapping function [32]. Linkage groups were assigned to chromosomes based on BC_1_ [33] and F_2_ [10] linkage maps and marker mapping information on the CottonGen database (http://www.cottongen.org/). The linkage groups were named as “ChrΧ” based on the linkage group length from long to short. QTL were identified using Windows QTL Cartographer version 2.5 (http://statgen.ncsu.edu/qtlcart/WQTLCart.htm) based on composite interval mapping (CIM). The statistical significance of the LOD threshold value was determined using a permutation procedure (1,000 times for all traits). The QTL nomenclature was adapted according to the method of McCouch et al. (1997) [34]. The resulting linkage map and QTL were drawn using MapChart V2.2 software [35].

### Meta-analysis of the co-located QTL

The meta-analysis of the co-located QTL was conducted using Biomercator V3 software (http://moulon.inra.fr/index.php/fr/component/docman/cat_view/21-logiciels/101-abi-project-and-software/104-biomercator-v21). A map file and a QTL file were required to import into the Biomercator V3 software [36]. The map file contains map name, marker name, and distance between adjacent makers, etc. And the QTL file contains map name, QTL name, chromosome name, trait, LOD score, phenotypic variance (PV), position of the QTL, etc. The order ‘meta-analysis’ was used to integrate the QTL to detect the hotspot region.

## Results

### Trait performance and correlation analysis in the RIL population

The traits of fiber qualities and yield components are summarized in S1 Table. Overall, the trait values of DH962 were higher than those of Jimian5 in fiber qualities, and Jimian5 demonstrated higher trait values than DH962 in yield components, except that no significant difference was observed for SI and LI. The RIL population performed transgressive segregation on all traits. The results of ANOVA, shown in S2 Table revealed that most of fiber quality and yield component traits presented significant genetic and environmental effects (P < 0.01), except that SI showed no significantly environmental effect. And the broad-sense heritabilities of the fiber quality and yield component traits were showed in Table 1. The genetic potential of fiber quality and yield component traits were general low in cotton [37–39]. Boll number had the lowest broad-sense heritability (16.19%), fiber length had the highest broad-sense heritability (70.82%).

**Table 1:** The broad-sense heritabilities of fiber quality and yield component traits in the RIL population.

Components of variation	FL	FU	MIC	FE	FS	SCW	LW	LP	SI	BN	LI
σ^2^	0.65	0.08	0.05	0.01	0.49	0.04	0.01	1.44	0.90	1.70	0.06
σ^2^_ge_	1.31	0.37	0.09	0.10	2.40	0.06	0.01	3.10	0.46	9.00	0.20
σ^2^_e_	1.15	1.13	0.12	0.21	2.80	0.23	0.04	6.60	0.38	70.00	0.53
H^2^_B_ (%)	70.82	35.96	70.41	18.77	47.22	62.37	67.59	61.21	58.21	16.19	19.13

σ^2^, the genotypic variance; σ^2^_ge_, the genotype and environment interaction variance; σ^2^_e_, the error variance; H^2^_B_, the broad-sense heritability

Genetic correlation analysis between fiber quality and yield component traits was calculated based on covariance (Table 2). FL was significantly and positively correlated with FS, FU, and significantly and negatively correlated with MIC, FE, LW, LP and BN. FS was significantly and positively correlated with FU and SI, and significantly and negatively correlated with MIC, FE, LW, LP, BN and LI. Among the yield component traits, most traits were positively correlated between two traits, except that SI was significantly and negatively correlated with LP.

**Table 2:** Genetic correlation analysis between fiber quality and yield component traits in the RIL population.

Trait	FL	FU	MIC	FE	FS	SCW	LW	LP	SI	BN
FU	0.68**									
MIC	-0.71**	-0.12								
FE	-0.59**	-0.53**	0.14							
FS	0.99**	0.98**	-0.52**	-0.91**						
SCW	-0.08	0.01	0.20**	0.09	-0.05					
LW	-0.45**	-0.10	0.46**	0.28**	-0.43**	1.00**				
LP	-0.76**	-0.26**	0.62**	0.41**	-0.82**	0.17*	0.97**			
SI	0.12	0.05	-0.05	-0.18*	0.19*	0.15	0.03	-0.18*		
BN	-0.21**	0.08	0.87**	0.62**	-0.70**	-0.04	0.43**	0.95**	0.28**	
LI	-0.51**	-0.09	0.47**	0.49**	-0.46**	0.71**	0.99**	0.98**	-0.01	0.86**

* Significance with P value of 0.05

** Significance with P value of 0.01

### Genetic map construction

**Fig 1:**
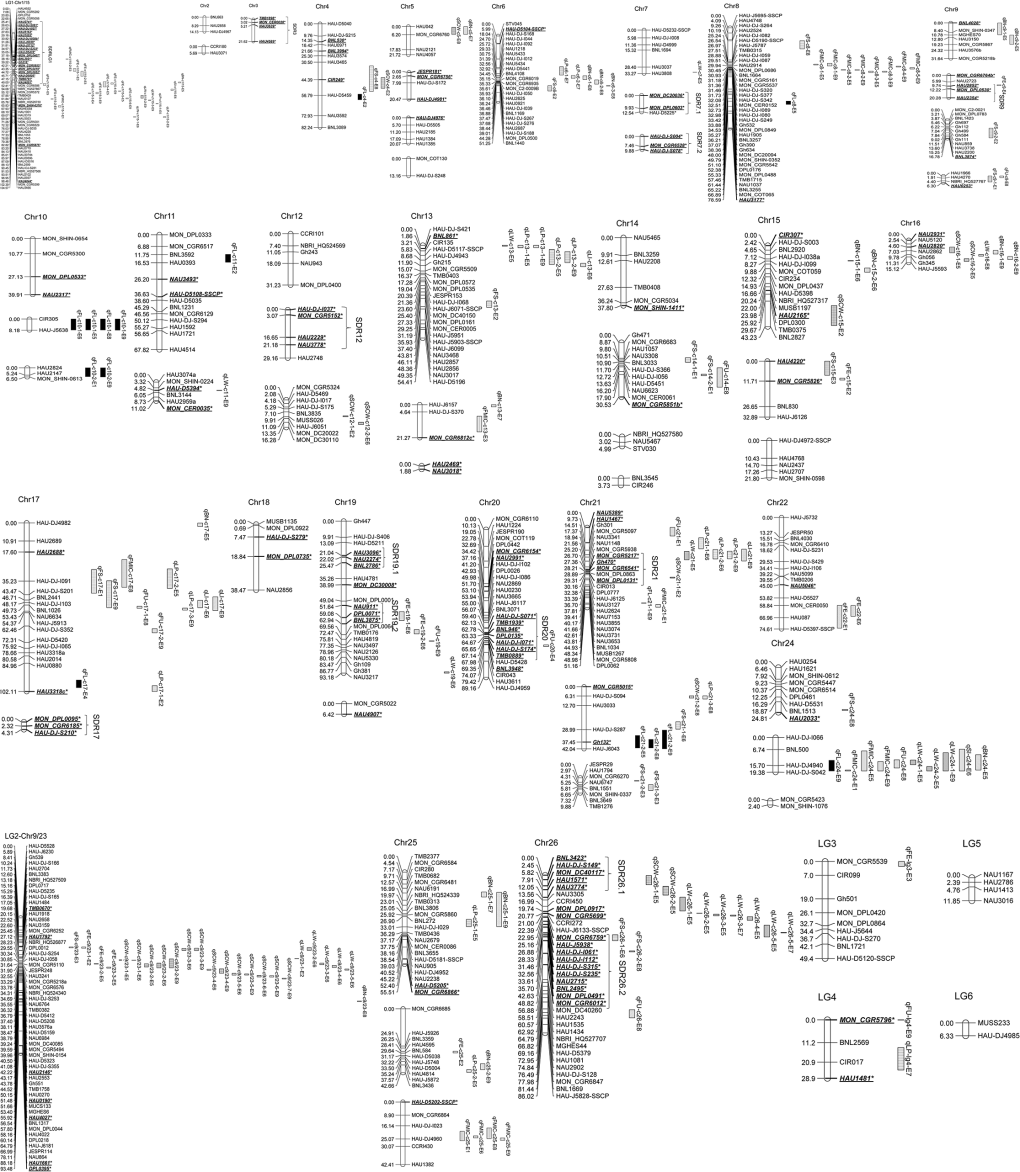
Genetic map and QTL for fiber quality and yield component traits in upland cotton. The map and QTL were detected under multiple environmental conditions in the RIL population derived from *G*. *hirsutum*. acc DH962 and *G*. *hirsutum*. cv Jimian5. The QTL were shown on the right of the Chromosomes/LGs. Markers showing segregation distortion are indicated as *asterisks* (*P < 0.05) and shown in *underlined*, *italic*, and *bold* text. The SDRs are shown in brackets.

A total of 644 loci were obtained from the selected 634 primer pairs from Wang et al. (2015) after genotyping the RIL population. After linkage analysis, 616 of 644 loci were mapped on 59 linkage groups; the total length of the linkage map was 2016.44 cM, with a mean distance of 3.27 cM between adjacent markers (Fig 1). The map included 538 SSR loci, 32 InDel loci and 46 SNP loci. Among the 59 linkage groups, there were 2–58 loci on each linkage group with 1.88–104.57 cM long. Fifty-three linkage groups were assigned to 24 chromosomes and 4 ones were unanchored; and most of the loci from two larger linkage groups (LG1-Chr1/15 and LG2-Chr9/23) were mapped on two pairs of homologous chromosomes (Chr1 and Chr15, Chr9 and Chr23, respectively).

### Marker distorted segregation

All the 644 loci were tested for the expected Mendelian 1:1 segregation ratio, and 144 (22.36%) loci showed a distorted segregation ratio (P < 0.05). Among these, 73 loci favored the female parent ‘DH962’ allele, and 71 loci favored the male parent ‘Jimian5’ allele. A total of 134 loci showing segregation distortion were mapped, and the distorted loci were unevenly mapped on different chromosomes. Thirteen segregation distortion regions (SDRs) were identified on 9 chromosomes and 1 linkage group (LG1-Chr1/15, Chr3, Chr7, Chr9, Chr12, Chr17, Chr19, Chr20, Chr21, and Chr26) (Fig 1). Most of the distorted loci in a given SDR skewed towards the same parent allele; for example, two large SDRs were located on LG1-Chr1/15 and Chr26, which showed significant distortion towards ‘DH962’ allele (Fig 1).

### QTL for fiber quality and yield component traits

A total of 134 QTL were detected on 21 chromosomes and 4 linkage groups, explaining 4.40–15.28% of the phenotypic variation (PV), with LOD scores ranging from 2.50 to 6.66 (S3 Table; Fig 1). In seven environments, 64 QTL for five fiber quality traits and 70 QTL for six yield components were identified. Among these QTL, 9 common QTL were detected in more than one environment. Among these 9 QTL, 5 QTL were associated with fiber qualities, and 4 QTL were associated with yield components. In addition, 22 of the 134 QTL and 19 new QTL (2 for FL, 1 for FS, 2 for MIC, 1 for FE, 3 for FU, 1 for SCW, 3 for LW, 2 for LP, 3 for BN, and 1 for LI) were identified in combined analysis (E9) (S3 Table).

#### Fiber length

Ten QTL were detected on 6 chromosomes (Chr4, Chr8, Chr10, Chr11, Chr17, and Chr21), with LOD scores ranging from 2.60 to 5.30, explaining 5.48–11.07% of the PV (S3 Table). Nine QTL derived from ‘DH962’ showed positive additive effects, and one QTL derived from ‘Jimian5’ showed a negative additive effect. The QTL qFL-c10-1 was detected in three individual environments (E5, E6 and E8), and this QTL, which was located between marker CIR305 and HAU-J5638, was also detected in combined analysis (E9), explaining 5.97–11.07% of the PV. The QTL qFL-c21-2 was detected in two environments (E5 and E8) and in combined analysis (E9), and the QTL qFL-c10-2 was detected in E1 and combined analysis (E9).

#### Fiber strength

A total of 18 QTL for fiber strength were identified on 11 chromosomes and 2 linkage groups (LG1-Chr1/15, Chr4, Chr6, Chr8, Chr9, LG2-Chr9/23, Chr13, Chr14, Chr15, Chr17, Chr21, Chr24, and Chr26), explaining 4.86–11.39% of the PV, with LOD scores ranging from 2.52 to 5.18 (S3 Table). Among these, 11 QTL showed positive additive effects originating from ‘DH962’, and 7 QTL showed negative effects originating from ‘Jimian5’. qFS-c17 was identified in E1 and combined analysis (E9), explaining 9.82 and 10.19% of the PV, with the LOD scores of 4.93 and 4.32, respectively.

#### Micronaire

Thirteen QTL were detected on 6 chromosomes and 1 linkage group (LG1-Chr1/15, Chr8, Chr13, Chr17, Chr21, Chr24, and Chr25), explaining 5.22–9.64% of the PV, with LOD scores ranging from 2.54 to 3.99 (S3 Table). The QTL qMIC-c24, originating from ‘Jimian5’, was identified in E1, E5 and combined analysis (E9), showing a negative additive effect and explaining 5.38% and 7.36% of the PV in E1 and E5, respectively. The QTL qMIC-c25 had a positive additive effect on MIC and was also detected in E1, E6, E8 and the combined analysis (E9). This QTL was located between the HAU-DJ-I023 and CCRI430 markers, explaining 5.80–7.20% of the PV. The QTL qMIC-c8-1, qMIC-c8-2 and qMIC-c8-3 were detected in both E5 and combined analysis (E9).

#### Fiber elongation

A total of 16 QTL were detected on 5 chromosomes and 3 linkage groups (LG1-Chr1/15, LG2-Chr9/23, Chr9, Chr15, Chr19, Chr22, Chr25, and LG3) (S3 Table). Among the 16 QTL, 9 QTL originated from ‘DH962’, and 7 QTL originated from ‘Jimian5’. The QTL qFE-c1/15-3 was identified in E4 and combined analysis (E9), and the QTL qFE-c1/15-4 was identified in E5 and combined analysis (E9). The QTL qFE-c22 was observed in E1 and E6, showing a negative additive effect on E1 and a positive additive effect on E6, explaining 11.92% and 7.79% of the PV with LOD scores of 4.96 and 3.10, respectively.

#### Fiber length uniformity ratio

Seven QTL were detected on 7 chromosomes (Chr9, Chr14, Chr17, Chr20, Chr21, Chr24, and Chr26), explaining 5.09–8.67% of the PV, with LOD scores ranging from 2.52 to 4.13 (S3 Table). Only one QTL was located in the A_T_ genome, and six QTL were located in the D_T_ genome. Among which, 5 QTL showed positive additive effects originating from ‘DH962’, and 2 QTL showed negative effects originating from ‘Jimian5’.

#### Seed cotton weight per boll

Nineteen QTL for SCW were detected on 7 chromosomes and 1 linkage group (Chr4, Chr5, LG2-Chr9/23, Chr12, Chr15, Chr16, Chr21, and Chr26), explaining 5.44–15.28% of the PV, with LOD scores ranging from 2.51 to 5.34 (S3 Table). Six QTL originated from the parent ‘DH962’, and 13 QTL originated from the parent ‘Jimian5’. The QTL qSCW-c9/23-1 and qSCW-c9/23-2 were observed in E5 and E6, and the QTL qSCW-c9/23-2 was also detected in combined analysis (E9). The QTL qSCW-c9/23-1 explained 5.57 and 5.74% of the PV, with LOD scores of 2.51 and 2.80, respectively. The QTL qSCW-c9/23-2 explained 5.92–7.82% of the PV, with LOD scores ranging from 3.20 to 3.99. Four QTL, qSCW-c9/23-3, qSCW-c9/23-4, qSCW-c9/23-5, and qSCW-c9/23-6, were detected in both E6 and combined analysis (E9).

#### Lint weight per boll

For lint weight per boll, 20 QTL were detected on 7 chromosomes and 2 linkage groups (LG1-Chr1/15, Chr7, LG2-Chr9/23, Chr13, Chr16, Chr19, Chr21, Chr24, and Chr26), explaining 4.40–9.93% of the PV, with LOD scores ranging from 2.50 to 4.70 (S3 Table). Ten QTL originating from ‘DH962’ showed positive additive effects on LW, and the other ten QTL originating from ‘Jimian5’ showed negative additive effects on LW. Two QTL, qLW-c26-3 and qLW-c26-5, were detected in both E5 and E7, both originating from the ‘DH962’ parent. The QTL qLW-c26-3 explained 9.36 and 9.48% of the PV, with LOD scores of 3.17 and 3.15, respectively. The QTL qLW-c26-5 explained 9.93 and 5.54% of the PV, with LOD scores of 3.58 and 2.53, respectively. qLW-c24-1 and qLW-c24-2 were identified in combined analysis (E9) as one QTL.

#### Lint percentage

A total of 13 QTL for LP were identified on 5 chromosomes and 2 linkage groups (LG1-Chr1/15, Chr6, Chr13, Chr17, Chr21, Chr25, and LG4), explaining 4.48–12.50% of the PV, with LOD scores range from 2.52 to 6.66 (S3 Table). Five QTL derived from ‘DH962’ showed positive additive effects, whereas eight QTL derived from ‘Jimian5’ showed negative additive effects. Two QTL, qLP-c13-1 and qLP-c13-2, were detected in both E5 and combined analysis (E9). The QTL qLP-c21-2 was detected in E6 and combined analysis (E9).

#### Boll number per plant

Fourteen QTL for BN were detected on 8 chromosomes and 2 linkage groups (LG1-Chr1/15, Chr5, Chr6, Chr9, LG2-Chr9/23, Chr13, Chr15, Chr17, Chr24, and Chr25), with LOD scores ranging from 2.52 to 4.07, explaining 5.15–9.08% of the PV (S3 Table). Eight QTL derived from ‘DH962’showed positive additive effects, whereas six QTL derived from ‘Jimian5’ showed negative additive effects. The QTL qBN-c25-1 was detected in E7 and combined analysis (E9).

#### Seed index and lint index

Two and two QTL were identified for SI and LI, respectively (S3 Table). Two QTL of SI explained 6.90 and 14.38% of the PV, with LOD scores of 3.49 and 6.64, respectively. Two QTL for LI explained 5.02 and 7.30% of the PV, with LOD scores of 2.69 and 3.71, respectively. The QTL qLI-c17 was detected in E6 and combined analysis (E9).

### QTL clusters

A total of 24 co-located QTL regions were observed on 13 chromosomes and 2 larger linkage groups (Chr4, Chr5, Chr6, Chr9, Chr10, Chr13, Chr14, Chr17, Chr21, Chr22, Chr24, Chr25, Chr26, LG1-Chr1/15, and LG2-Chr9/23). After QTL meta-analysis, 26 QTL hotspot regions were obtained (S4 Table; S1 Fig). Except for 9 common QTL, 17 QTL hotspot regions affected different fiber quality or yield component traits (S1 Fig). For example, on LG2-Chr9/23, there were 3 QTL related to different traits (FS, FE, and LW) distributed on a region of 0.46 cM (S4 Table; S1 Fig). Several QTL clusters were distributed on 15 chromosomes and 2 linkage groups (Chr4, Chr5, Chr6, Chr8, Chr9, Chr10, Chr13, Chr14, Chr16, Chr17, Chr21, Chr22, Chr24, Chr25, Chr26, LG1-Chr1/15, and LG2-Chr9/23). On LG2-Chr9/23, 18 QTL included 4 traits (FS, FE, SCW, and LW) were located on a region ranging from 20.1 to 40.8 cM. Among the 18 QTL, 8 QTL were associated with SCW, 5 QTL were associated with LW, 3 QTL were associated with FE, and 1 QTL was associated with FS.

## Discussion

In the present study, 616 loci were mapped on 59 linkage groups, and the total length of the linkage map was 2016.44 cM, covering 40.33% of the upland cotton genome. Compared with high-density interspecific genetic maps [40–43], the intraspecific genetic map generated in the present study showed low coverage throughout the cotton genome. This phenomenon was also observed for the construction of other genetic maps in upland cotton [11,13,14]. Due to the narrow genetic base of upland cotton, we screened most of the SSRs in the CottonGen database (http://www.cottongen.org) and some SNPs and InDels developed in the laboratory [15], but the results were not satisfactory, and with the increase in map density, the efficiency of QTL detection was greatly improved [7,15]. There is no high-density and coverage genetic map for upland cotton, which is the huge obstacle for QTL mapping in upland cotton. In previous studies [14,15], the effect of SSRs was lower in the construction of the upland cotton genetic map, and the development of lots of SNPs and InDels using next-generation sequencing technology could provide a new outlet for future studies.

Segregation distortion widely exists in the study of population genetics. In the present study, 144 (22.36%) of the 644 loci showed segregation distortion. In previous studies of cotton [3,8,11,13,14,19,27,42,44–50], many reports have focused on segregation distortion, we summarized these results in S5 Table. The percentages of distorted loci in interspecific populations ranged from 7.65% to 25.50%, while they ranged from 22.36% to 81.25% in intraspecific populations. Although the genetic difference is bigger, the segregation distortion is smaller in interspecific populations, and this phenomenon presents an interesting topic for future studies. S5 Table also showed that most of the distorted loci skewed toward upland cotton in interspecific populations, and most of the distorted loci skewed toward one of the parents in upland cotton in intraspecific populations. Lacape et al. (2010) showed that the average fiber characteristics of the interspecific RILs were closer to the *G*. *hirsutum* parent than to the *G*. *barbadense* parent, and 71% and 29% of the observed distorted allelic skewed toward *G*. *hirsutum* and *G*. *barbadense*, respectively. As the important cultivated cotton, most of the varieties of upland cotton were improved, and some good genes from other *Gossypium* species were introgressed into upland cotton through hybridization and backcross methods. The introgression of good genes generated the genetic differences observed between the mapping parents of upland cotton, resulting in segregation distortion [8,11,13,14,19]. The number of distorted loci skewed toward ‘DH962’ and ‘Jimian5’ were similar, and the percentage of distorted loci was lower than that in other upland cotton populations in the present study, suggesting that the ability of selection and the combination of gametophyte mapping parents are similar. In addition, the fiber quality and yield component traits were significantly different (S1 Table), and all the results showed that the two parents were suitable for the development of the RIL population.

The clustering of QTL in tetraploid cotton has been reported in some studies [3,8,11,13,14,19,20,47,51,52]. The present study also identified 26 QTL hotspot regions, among which 17 QTL hotspot regions affected two or more different fiber quality or yield component traits. The phenomenon of QTL clustering might represent the linkage of genes and QTL or result from pleiotropic effects of a single QTL in the same genomic region [3]. For example, NAU5107, detected on E5, was the nearest marker of qFS-c1/15-1 and qFE-c1/15-4; and FS was significantly and negatively correlated with FE in the present study (Table 2). These QTL hotspot regions revealed that the linkage drag of QTL hindered the improvement of fiber quality [20]. In addition, 11 larger QTL clusters were also obtained in the present study. As shown in Fig 1, we observed that most of the clusters showed the enrichment of fiber quality or yield component traits. On LG1-Chr1/15, most of the QTL were correlated with FS and FE in the cluster. On Chr13, most of the QTL were correlated with LP and BN in the cluster. On LG2-Chr9/23, 18 QTL were distributed on the QTL clusters, 8 QTL were associated with SCW, and 5 QTL were associated with LW. Previous studies [51,52] generated multiple QTL clusters and hotspots of fiber quality or yield component traits in the cotton genome through the analysis of most of the publications on cotton. The QTL clusters provided valuable information to determine genome regions with different traits.

S2 Table showed that all the fiber quality and yield component traits presented significant environmental effects, and the present study revealed that cotton traits were highly affected by environments and climate conditions. An example of meteorological information of E5, E6, E7 and E8 is shown in S6 Table and S2 Fig. S6 Table showed that the number of rainy day during the four years were different. For example, 14 rainy days were reported in E7, but 6 rainy days were reported in E8 in August. And S7 Table showed that the temperature presented significant environmental effects (P < 0.01) in July and August, and these two months represented a critical period for blossom and fiber development in cotton. The different climate conditions during the two months might have seriously affected the fiber quality and yield of cotton.

Because the genetic maps were constructed based on different populations and genetic backgrounds, and environmental elements would affect the expression of QTL, it was difficult to obtain common QTL among different populations. However, lots of QTL observed in the present study were still identified according to the same markers of the same chromosomes in other reports. The QTL qLP-c6-2 was detected as QTL-lp2 in the F_2_ population from a cross between TM-1 and T586 [53]. QTL qFS-c14-1 corresponded to the QTL qFS-D2-1 in the 4WC population [20]. QTL qFE-c1/15-2, qFE-c9/23-1, qFL-c17, qFS-c9/23, and qFU-c17-1 were detected as qFE-A1-1, qFE-A9-1, qFL-D3-1, qFS-A9-1, and qFU-D3-1, respectively, in the RIL population resulting from a cross between Prema and 86–1 [13]. The QTL qFL-c10-2 was detected as the major QTL qFL10.1 in the RIL population resulting from a cross between the upland cotton cultivars Yumian 1 and 7235 [14]. QTL qFL-c10-2 was also detected as a major QTL qFL10.1 in the report of Shao et al (2014); and qFL-c8, qFMIC-c1/15-1, qFMIC-c8-5, qFE-c25, qFS-c9-2, and qFS-c21-3 were detected as qFL08.1, qFM01.1, qFM08.1, qFE25.1, qFS09.1, and qFS21.2 in this research [25], respectively. The QTL qFMIC-c1/15-2 was detected as qMC-07A-c1-1 in an interspecific population [3]. And qFE-c15 was the same as the QTL qFE15.2 in a three-parent composite population of upland cotton [12]. QTL qFE-lg3 and qFE-c22 were detected as qELO-c18-2 and qELO-c22 in a random-mated recombinant inbred population [54], respectively. At the same time, qBN-c6-1 was detected as qNB-A6-1 by linkage analysis and association mapping [55]. The QTL qLP-c13-1 and qMIC-c24 were detected as qLP-c13 and qMV-c24, respectively, in a F_2_ population based on the same parents used in the present study [15]. These QTL across different populations could be examined to obtain candidate genes of related traits, and the adjacent markers of these QTL could be used for marker-assisted selection. Some common makers (BNL2495, BNL4028, BNL4108, Gh584, HAU2147, MON_CGR5826, MON_CGR6019, MON_CGR6764, MON_SHIN0613, NAU2820, NAU3774, NAU3308, NAU3948, NAU4891, and NAU5433) were also identified in other cotton studies [3,9,12–15,20,48,53,56]. For example, BNL4108 was associated with BN and LP in the present study, but associated with FL, FU, FE, and LP in previous studies [9,53]. NAU3308 was associated with FS in the present study, but associated with FL, FS, SI, and LP in a previous study [20]. MON_CGR5826 was associated with FE and FS, but associated with FU in the report of Zhang et al. (2012) [12]. The common markers associated with different traits might result in the development of QTL clusters and linkage drag.

Among the 134 QTL detected in the present study, 9 common QTL were obtained in more than one environment. The difficulty of obtaining stable QTL has been reported in the previous studies [8,11,12,14]. Two QTL, qFL-c10-1 and qMIC-c25, were detected in three environments and combined analysis (E9), and the results revealed that fiber quality traits were more stable than yield traits under multiple environmental conditions. Notably, two stable QTL were not clustered with any other QTL associated with fiber quality and yield traits, providing an opportunity to identify candidate genes for improving the fiber quality of upland cotton.

## Supporting Information

S1 Fig QTL Meta-analysis of hotspot regions using BiomercatorV3 software. (TIF)

S2 Fig Average temperatures in July, August and September in E5, E6, E7 and E8. (TIF)

S1 Table Phenotypic value of fiber quality and yield component traits in the RIL population and the parents. (XLS)

S2 Table Variance analysis of fiber quality and yield component traits in the mapping population. (XLS)

S3 Table QTL for fiber quality and yield component traits in the RIL population under multiple environmental conditions. (XLS)

S4 Table The AIC scores of the QTL hotspot regions analyzed by Biomercator V3 software. (XLS)

S5 Table Characteristics of distorted loci in interspecific and intraspecific populations. (XLS)

S6 Table Weather characteristics in July, August and September in E5, E6, E7 and E8. (XLS)

S7 Table Variance analysis of the average temperatures in July, August and September in E5, E6, E7 and E8. (XLS)
