# Supplementary figures and images for: QTL Mapping for Fiber and Yield Traits in Upland Cotton under Multiple Environments
Source: PLoS One. 2015 Jun 25;10(6):e0130742. doi: 10.1371/journal.pone.0130742 (PMC4481505; doi:10.1371/journal.pone.0130742)

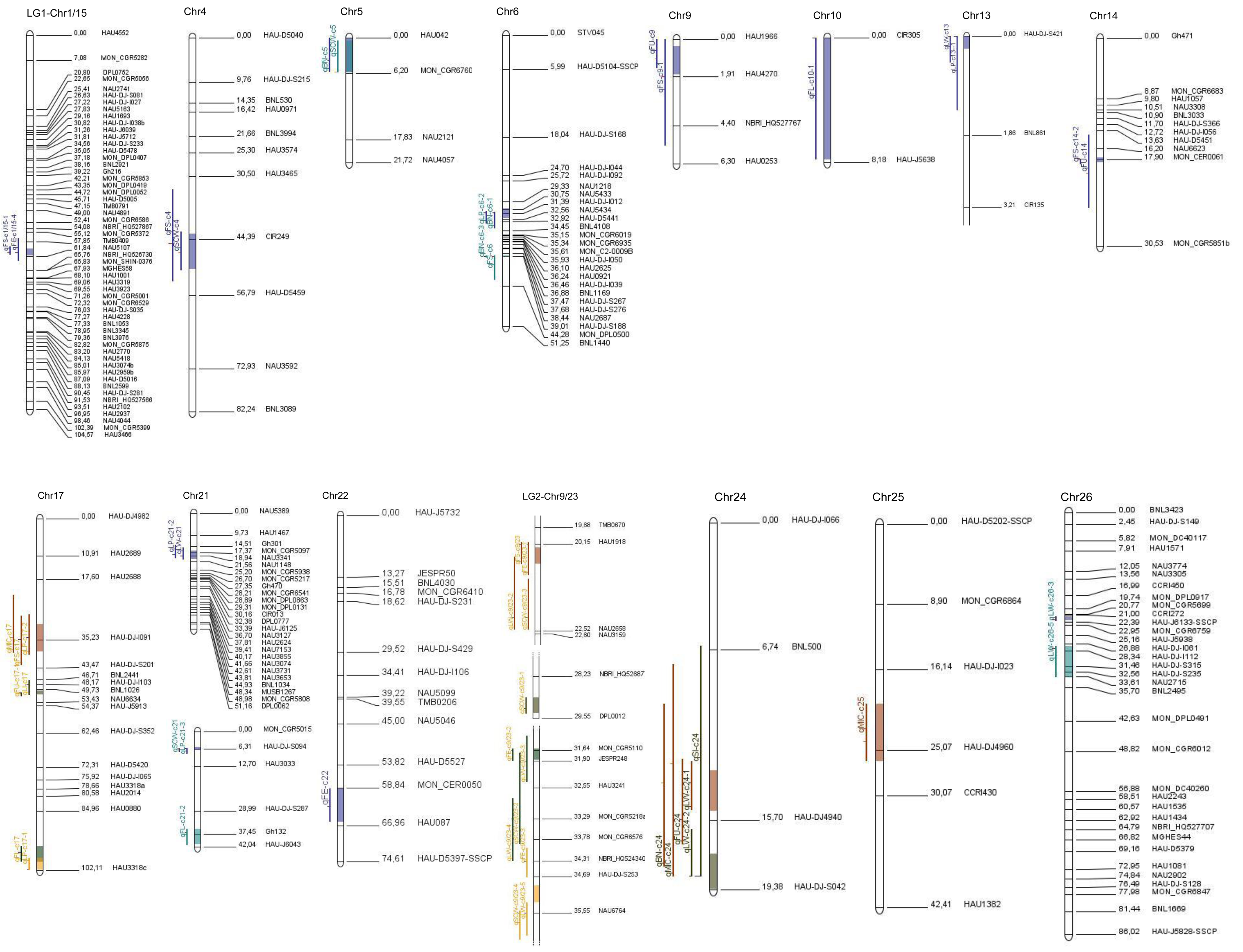

Supplement: S1 Fig — (TIF) [file pone.0130742.s001.tif]

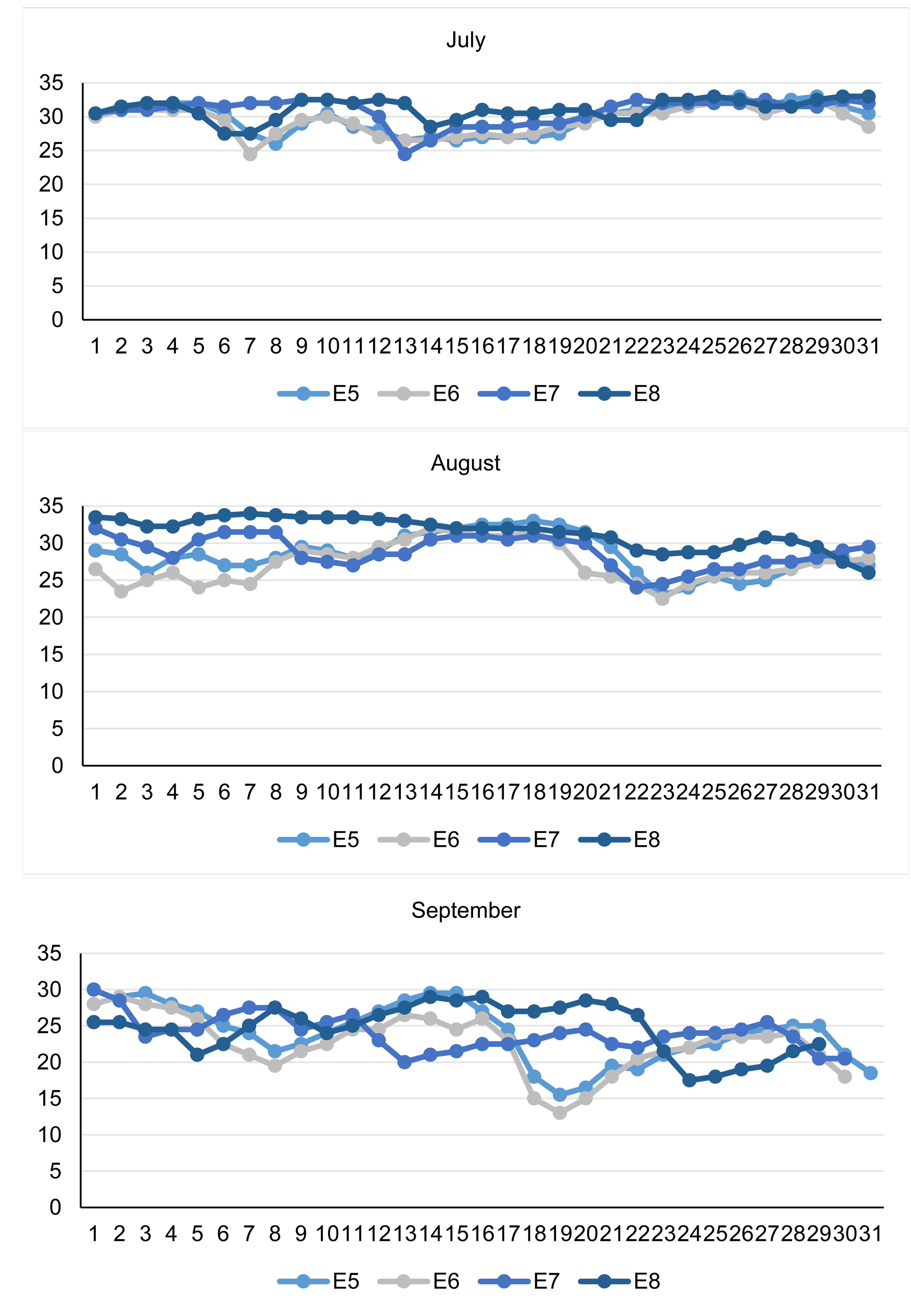

Supplement: S2 Fig — (TIF) [file pone.0130742.s002.tif]
